# Editorial: Postprandial physiology

**DOI:** 10.3389/fnut.2022.1107480

**Published:** 2022-12-09

**Authors:** Jarlei Fiamoncini, John Newman, Lorraine Brennan

**Affiliations:** ^1^Food Research Center (FoRC), Department of Food Science and Experimental Nutrition, School of Pharmaceutical Sciences, University of São Paulo, São Paulo, Brazil; ^2^Western Human Nutrition Research Center, Agricultural Research Service, United States Department of Agriculture (USDA), Davis, CA, United States; ^3^Department of Nutrition, University of California, Davis, Davis, CA, United States; ^4^West Coast Metabolomics Center, Genome Center, University of California, Davis, Davis, CA, United States; ^5^Institute of Food and Health, School of Agriculture and Food Science, Conway Institute, University College Dublin, Dublin, Ireland

**Keywords:** postprandial, oral glucose tolerance test (OGTT), mixed meal tolerance test (MMTT), postprandial inflammation, nutritional physiology

There is growing interest in understanding responses to food and nutrient intake for the study of health and disease. This interest has been triggered in part by the involvement of physiological processes that take place following the ingestion of a meal in the etiology of chronic diseases and to recent findings that have brought new perspectives to postprandial metabolism (1). For instance, the causes, consequences, and physiological role of postprandial inflammation has gained much attention and may ultimately provide unique understanding of vascular and metabolic disease risks and progression (2–4). Similarly, since the discovery of bile acid (BA)-sensitive receptors over 20 years ago, several functions not fully understood have been attributed to these metabolites in addition to their long established role in the digestion of lipids (5). In general, probing the postprandial dynamics in metabolite concentrations may provide novel diagnostic opportunities. Similar to changes in glucose and insulin levels during an oral glucose tolerance test (OGTT), changes circulating levels of other metabolites following a meal can often reveal metabolic dysregulation or the effects of an intervention with greater sensitivity than the plasma concentration of such markers in the fasted state (6, 7).

One of the biggest challenges to the study of postprandial metabolism is the large interindividual variability observed in the metabolic responses to food intake. Such variability is no surprise as numerous factors including gastric emptying, intestinal transit, food digestion, and nutrient absorption, as well as the secretion of signaling molecules in response to the meal and their effects in target tissues play along in the orchestrated events that define systemic responses to food intake. Despite the complexity associated with the high interindividual variability, understanding its causes will constitute a great advance toward the development of personalized nutrition. Matching the challenges associated with the study of postprandial metabolism, scientists can now count on tools such as metabolomics and genomics, that are evolving and readily available to numerous groups, enabling novel observations that often challenge concepts of physiology laid decades ago. To get an idea of the impact of these novel technologies, 7 out of 8 articles in Research Topic issue come from studies that employed such tools.

From this issue, Weinisch et al. compared the metabolic responses of a group of young males to three different dietary challenges, using 600 metabolites profiled using metabolomic platforms. A core set of metabolites were identified as responding to all three challenges. The results indicate what are the hot targets if one is interested in assessing adaptations to the intake of macronutrients. From the same HuMet study, Fiamoncini, Rist et al. focused exclusively on the dynamics of plasma BA after different metabolic challenges that include an extended fasting and two postprandial tests. The study highlights the high interindividual variability of BA appearance in plasma as well as the large amplitude of this phenomenon. The biggest novelty of the report was the description of decreased BA levels in response to 36 h of fasting.

Fiamoncini, Donado-Pestana et al. also contributed to this issue with an article based on data generated during the NutriTech study. Considering only glucose concentrations measured during an OGTT, two groups of individuals were identified amongst the study population with clearly different insulin sensitivity, even though all subjects were considered euglycemic according to WHO guidelines for fasting glucose levels. The study confirms previously published markers of insulin resistance/sensitivity and indicates the association of specific metabolites with better glycaemic responses during the OGTT.

Hedbäck et al. contributed with a report described the effects of meal texture (solid × liquid) to postprandial glycaemia and incretin levels. Examining a cohort of patients that underwent bariatric surgery, the authors report no effects of the meal texture on postprandial glycemia, despite small differences in the concentration of incretins. In the patients following bariatric surgery, the liquid meal elicited higher GLP-1 levels in the 1^st^ h after food intake. The study is an important contribution and validates the use of liquid meals to study postprandial metabolism, regardless of bariatric surgery.

Newman et al. report the combined results of 2 studies, concluding that the execution of a mixed meal tolerance test (MMTT) can simultaneously inform on an individuals' insulin sensitivity and postprandial lipid handling. After conducting MMTTs in over 300 subjects from both genders and a broad range of age and BMI, the authors document the high heterogeneity in postprandial lipemic response identifying four patterns of response to the test meal with an assessment of the response stability over time and providing insights linking insulin resistance and dysregulated lipid metabolism. Also examining the postprandial appearance of triglycerides following a meal challenges (oral lipid tolerance test), Alcala-Diaz et al. described the association between a common genetic variant in the ZPR1 gene and a dietary intervention. The results highlight the importance of gene × diet interaction in the promotion of health outcomes.

This issue also brings a contribution from Kim et al. that employed a dietary challenge with yoghurt and milk to identify food intake. The authors described several metabolites associated to each test food and identified markers sensitive to the interaction between age and food intake. Postprandial challenge tests remain a key tool in the development of new food intake biomarkers in an effort to improve accuracy of dietary assessment.

Finally, the article from Schlicker et al. addresses the potential of flux analysis when assessing postprandial metabolism. Using either a bolus of glucose or a wheat protein meal, both equivalent in terms of carbohydrate content and glucose labeling, the authors highlight the role of lactate as a “metabolic buffer” that helps keeping plasma glucose levels regulated after the intake of a meal. The application of flux analysis during postprandial challenges needs further development and warrants interesting discoveries.

This collection of articles displays a range of applications of postprandial challenges and highlights their potential for the development of new knowledge to better characterize the physiological perturbations associated to disease risk and progression. The results indicate that harnessing the individual responses to meal challenges is likely to play a key role in the development of precision nutrition.

## Author contributions

All authors listed have made a substantial, direct, and intellectual contribution to the work and approved it for publication.
